# Correlation between maximal tumor diameter of fresh pathology specimens and computed tomography images in lung adenocarcinoma

**DOI:** 10.1371/journal.pone.0211141

**Published:** 2019-01-25

**Authors:** Chul Hwan Park, Tae Hoon Kim, Sungsoo Lee, Duk Hwan Moon, Heae Surng Park

**Affiliations:** 1 Department of Radiology, Research Institute of Radiological Science, Gangnam Severance Hospital, Yonsei University College of Medicine, Seoul, Korea; 2 Department of Thoracic and Cardiovascular Surgery, Gangnam Severance Hospital, Yonsei University College of Medicine, Seoul, Korea; 3 Department of Pathology, Ewha Womans University Mokdong Hospital, Seoul, Korea; Toranomon Hospital, JAPAN

## Abstract

The authors compared maximal tumor diameters between fresh lung tissue and axial and multiplanar reformatted chest computed-tomography (CT) images in lung adenocarcinoma and investigated the factors affecting tumor-size discrepancies. This study included 135 surgically resected lung adenocarcinomas. An experienced pulmonary pathologist aimed to cut the largest tumor section and measured pathological tumor size (PTS) in fresh specimens. Radiological maximal tumor sizes (RTS) were retrospectively measured on axial (RTSax) and multiplanar reformatted (RTSre) chest CT images. Mean PTS, RTSax, and RTSre were 19.13 mm, 18.63 mm, and 20.80 mm, respectively. RTSre was significantly larger than PTS (mean difference, 1.68 mm; *p*<0.001). RTSax was also greater than PTS for 6−10-mm and 11−20-mm tumors. PTS and RTS were strongly positively correlated (RTSax, r^2^ = 0.719, *p*<0.001; RTSre, r^2^ = 0.833, *p*<0.001). The intraclass correlation coefficient was 0.915 between PTS and RTSax and 0.954 between PTS and RTSre. Postoperative down-staging occurred in 11.0% and 27.4% of tumors on performing radiological staging using RTSax and RTSre, respectively. Postoperative up-staging occurred in 12.3% and 1.4% of tumors on performing radiological staging using RTSax and RTSre, respectively. Multiple linear regression revealed that pleural dimpling (*p* = 0.024) was an independent factor affecting differences between PTS and RTSax. Specimen type (*p* = 0.012) and tumor location (*p* = 0.020) were independent factors affecting differences between PTS and RTSre. In conclusion, RTSre was significantly larger than PTS and caused postoperative down-staging in 27.4% of the tumors. Reliability analysis revealed that RTSre was more strongly correlated with PTS than RTSax. Specimen type and anatomical tumor location influenced the measured size differences between PTS and RTSre.

## Introduction

Tumor size is an important prognostic factor in cancers of solid organs. There are, however, discrepancies in tumor size measured using pathological and radiological methods. In renal tumors, computed tomography (CT) of radiological tumor size (RTS) generally overestimates pathological tumor size (PTS)[1, 2]. In non-small cell lung cancer (NSCLC), chest CT usually overestimates PTS[3–5]; however, conflicting results have been reported[6]. Lung tumor size discrepancies between radiological and pathological methods are affected by several factors. First, the degree of lung aeration and expansion when measuring PTS and RTS is quite different. In lung cancer, RTS is usually measured in the fully expanded condition when the patient holds a deep breath. However, PTS is measured with the lung in the collapsed condition, when it is compressed during one-lung ventilation for surgery, and the resected lung tissue becomes more flat due to deflation and blood drainage after removal of surgical clip(s) or staples(s)[3, 5]. Second, the tumor planes in CT and specimens, where the maximal tumor size is measured, are different[3]. For example, radiologists usually measure tumor size in the axial plane of chest CT, unless the tumor extends vertically lengthwise. However, pathologists tend to cut the largest tumor section perpendicular to the visceral pleura and resected surface to assess pleural invasion and resection margin, and then measure tumor size. If the tumor shape is vertically long, the axial plane of chest CT may underestimate tumor size compared with sagittal or coronal planes of CT, or pathology specimen.

Further, PTS of the lung can change during routine pathology processes such as formalin fixation[7], tissue processing, and slide preparation[8]. Formalin fixation can shrink tumor size and cause down-staging in 3%-10% of the specimens from patients with NSCLC[7, 9]. Hsu *et al*. reported that fresh tumor size was more strongly related to patient prognosis than fixed tumor size in stage I NSCLC[9]. To date, radiological and pathological correlations of lung tumor size have been assessed using RTS entirely measured on axial CT images[5, 6, 8, 10–12], or PTS measured on fixed specimens[4, 6, 11] or on glass slides (i.e., microscopic measurement)[3]. Therefore, we compared maximal tumor diameters between fresh pathology specimens and CT images (axial and multiplanar reformatted images) in lung adenocarcinoma, and investigated the factors that influence tumor size discrepancies between pathological and radiological methods.

## Materials and methods

### Case selection and measurement of PTS

**Fig 1:**
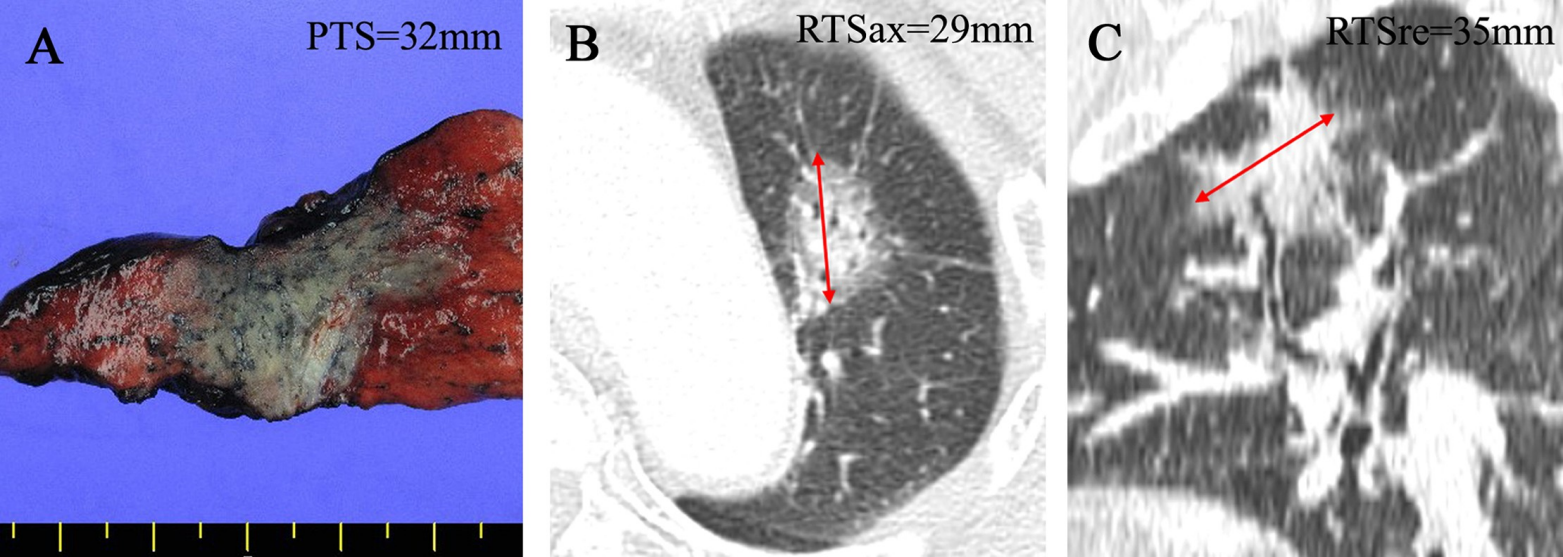
Measurement of pathological and radiological tumor sizes (RTS). (A) Pathological tumor size (PTS) was measured in fresh specimens after cross-sectioning. (B) First, RTS was measured on the axial plane (RTSax). (C) Multiplanar reformatted images were then reconstructed to define the greatest diameter of the lesion in three dimensions and the largest diameter of the lesion (RTSre) was measured.

The cases included in this study were adopted from a prospectively collected lung cancer data-base for another prospective study[7]. The data-base included surgically resected non-small cell lung cancer tissues (n = 200) that were sectioned and measured in the fresh state at the Department of Pathology, Gangnam Severance Hospital (Seoul, South Korea) between 2013 and 2016. From the data-base, adenocarcinoma was selected for this study. The specimens were not inflated with embedding medium or fixatives before gross cutting; PTS was measured exclusively in the fresh state. An experienced pulmonary pathologist (H.S.P.) aimed to cut the largest tumor section, usually in the center of the tumor mass, and measured the maximal tumor diameter using a straight metal ruler (Fig 1A); PTS was measured including both solid and subsolid portions of the tumor mass. Tumors larger than 5 cm, those with uncertain margins owing to underlying lung fibrosis or pneumonia, and those that exhibited larger cross-sectional diameter after formalin fixation were excluded. The initial gross measurement was re-evaluated at the time of microscopic evaluation of the tumor and none of the tumors required significant revision of the grossly estimated tumor size. Histopathological features, including specimen type containing tumor, tumor location (upper, middle, and lower lobe), ischemic time, gross type (solid, subsolid, mucinous) in pathology specimens, pleural invasion, pleural dimpling, tumor necrosis, adenocarcinoma classification according to the 2015 World Health Organization classification[13], and major histological pattern (lepidic versus vs. non-lepidic) were evaluated. Major histological pattern was defined as the most predominant histologic pattern of the tumor mass. In this study, minimally invasive adenocarcinoma and adenocarcinoma in situ were considered to exhibit lepidic dominant pattern. The histopathological features were defined as previously described[7]. Slice thickness in CT was also evaluated. Patient outcome was investigated until the date of death or last follow-up. The study protocol was approved by the Institutional Review Board of Gangnam Severance Hospital (protocol no: 3-2017-0319) and the requirement for informed consent was waived.

### CT protocol and image analysis

All chest CT scans were performed using one of the following three scanners: Somatom Sensation 16, Somatom Sensation 64, or Somatom Definition AS+ (all from Siemens Medical Solutions, Erlangen, Germany). Images were acquired from the lung apex to the adrenal glands, during breath-holding at the end of inspiration with a helical technique. Chest CT parameters included tube voltage, 120 kVp; tube current, 50–130 mA; and slice thickness, 1–3 mm. The data were reconstructed at 1–3 mm intervals on the scanner workstation. All CT images were uploaded to a commercially available reconstruction program (Aquarius iNtuition, version 4.4.12; TeraRecon, Foster City, CA, USA) for tumor size measurements.

A radiologist with >10 years’ experience in chest radiology measured RTS to one decimal place (C.H.P.), and was blinded to the results of pathological size measurements. RTS was measured including both solid and subsolid/ground glass opacity parts. For the RTS measurement, a lung window setting was used (width, 1500 Hounsfield units [HU]; level, –500 HU). First, the maximal diameter of the lesion in the axial plane (RTSax) was measured using an electronic caliper (Fig 1B). All CT slices containing each tumor were reconstructed in coronal, sagittal, and oblique planes by a radiologist (C.H.P.), and the maximal tumor diameter was carefully measured in each plane. Subsequently, the largest tumor diameter among multiplanar reformatted images (RTSre) was selected for analysis (Fig 1C).

### Statistical analysis

The paired *t*-test was used to compare mean PTS, RTSax, and RTSre. Scatter plots were used to determine relationships between PTS and RTS. Inter-observer reliability between PTS and RTS was analyzed by calculating the intra-class correlation coefficient (ICC) and performing Bland-Altman analysis. Radiological and histopathological factors affecting differences between PTS and RTS were assessed using the independent *t*-test, and stepwise multiple linear regression. Cancer-specific and progression free survival was estimated using the Kaplan-Meier method and compared using the log-rank test.

All statistical analyses were performed using SPSS version 17.0 (IBM Corporation, Armonk, NY, USA) and MedCalc version 14.8.1. (MedCalc Software, Ostend, Belgium) for Windows (Microsoft Corporation, Redmond, WA, USA). A *p*-value <0.05 was considered statistically significant.

## Results

A total of 135 tumors from 128 patients were included in the study. Mean time interval between CT scan and surgery was 17 days (range, 0–91). Demographic data from the study population are summarized in Table 1. Mean (± standard deviation) PTS, RTSax, and RTSre were 19.13±9.41 mm, 18.63±8.60 mm, and 20.80±9.46 mm, respectively. Mean RTSre was significantly larger than PTS (mean difference, 1.68±3.94 mm; *p*<0.001); however, there was no significant difference between RTSax and PTS (mean difference, 0.49±5.31 mm; *p* = 0.285). When tumors were divided into 10-mm intervals, the mean RTS (both RTSax and RTSre) was significantly greater than the mean PTS for tumors in 6–10 mm and 11–20 mm categories, and the mean difference was greater in RTSre than in RTSax (Table 2). For tumors in the 21–30 mm category, RTSax was significantly larger than PTS. However, PTS was significantly larger than RTSax for tumors in the 41–50 mm category. In the remaining categories, there was no significant difference between PTS and RTS.

**Table 1:** Baseline characteristics of lung adenocarcinoma (n = 135).

Parameter	
Age (year)	
Mean ± SD	62.5±10.7
Range	30–90
Sex	
Male	64 (47.4%)
Female	71 (52.6%)
Pathological tumor size (mm)	
Mean ± SD	19.1±9.4
Range	6–45
Radiological tumor size (mm) measured on the axial image	
Mean ± SD	18.8±8.5
Range	5.9–47
Radiological tumor size (mm) measured on the multiplanar reconstructed image	
Mean ± SD	20.8±9.5
Range	5.9–48.4
Specimen type containing tumor	
Wedge resection	71 (52.6%)
Segmentectomy	10 (7.4%)
Lobectomy	53 (39.3%)
Pneumonectomy	1 (0.7%)
Ischemic time (h)	
≤24	119 (88.1%)
>24	16 (11.9%)
Histologic classification	
Invasive adenocarcinoma	120 (88.9%)
Minimally invasive adenocarcinoma	12 (8.9%)
Adenocarcinoma in situ	3 (2.2%)
Tumor location	
Upper lobe	80 (59.3%)
Middle lobe	7 (5.2%)
Lower lobe	48 (35.5%)
Gross type of tumor in pathology specimen	
Solid	75 (55.6%)
Subsolid	55 (40.7%)
Mucinous	5 (3.7%)
Pleural invasion	
Absent	111 (82.2%)
Present	24 (17.8%)
Pleural dimpling	
Absent	81 (60%)
Present	54 (40%)
Tumor necrosis	
Absent	133 (98.5%)
Present	2 (1.5%)
Pathological TNM stage^a^	
0	3 (2.2%)
I	113 (83.7)
II	8 (5.9%)
III	8 (5.9%)
Recurrent	3 (2.2%)

^a^Stages adopted from the American Joint Committee on Cancer TNM staginig system, 8^th^ edition

SD, standard deviation; TNM, tumor, node, metastasis

**Table 2:** Mean pathological and radiological tumor sizes divided into 1-cm intervals by pathological tumor size.

PTS (mm)	n	Mean PTS±SD (mm)	Mean RTSax±SD (mm)	Mean RTSre±SD (mm)	Mean difference		*p*-value	
					PTS-RTSax^a^±SD (mm)	PTS-RTSre^b^±SD (mm)	a	b
≤10	30	8.4±1.5	9.6±3.2	10.5±3.4	-1.20±2.8	-2.1±2.9	0.024	<0.001
>10 but ≤20	52	15.5±2.5	16.5±4.9	17.9±2.6	-1.1±3.6	-2.4±3.5	0.035	<0.001
>20 but ≤30	37	24.9±2.2	23.3±4.1	25.8±4.1	1.6±4.2	-0.9±4.0	0.023	0.174
>30 but ≤40	10	33.9±2.4	34.2±6.8	35.9±6.4	-0.3±6.2	-2.0±6.2	0.902	0.342
>40 but ≤50	6	44.0±1.3	31.0±8.2	41.7±5.0	13.0±8.0	2.3±5.6	0.028†	0.345‡

†,‡: *p* values were calculated using the Wilcoxon signed rank test.

PTS, pathological tumor size; RTSax, radiological tumor size measured on axial images; RTSre, radiological tumor size measured on multiplanar reformatted images; SD, standard deviation

**Fig 2:**
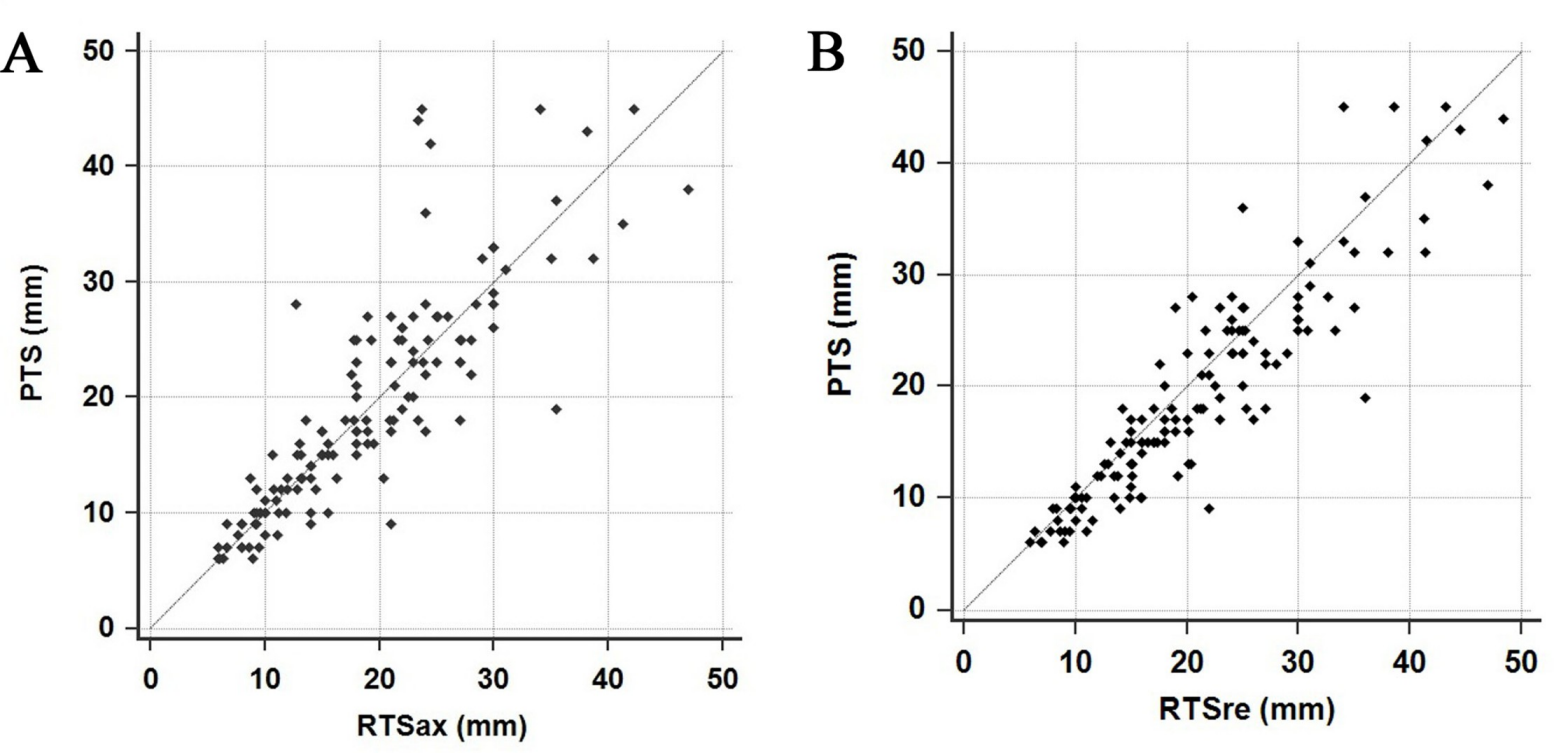
Scatter plots of pathological tumor size (PTS) vs. radiologic tumor size (RTS). (A) The relationship between PTS and RTS measured on axial images (r^2^ = 0.719, *p*<0.001). (B) The relationship between PTS and RTS measured on reconstructed multiplanar images (r^2^ = 0.833, *p*<0.001).

Scatter plots of PTS vs. RTSax and RTSre are shown in Fig 2; PTS was relatively strongly correlated with RTSax (r^2^ = 0.719, *p*<0.001) and RTSre (R^2^ = 0.833, *p*<0.001). Further, RTSax tended to overestimate PTS in small tumors, but underestimated PTS in larger tumors; RTSre tended to consistently overestimate PTS.

**Fig 3:**
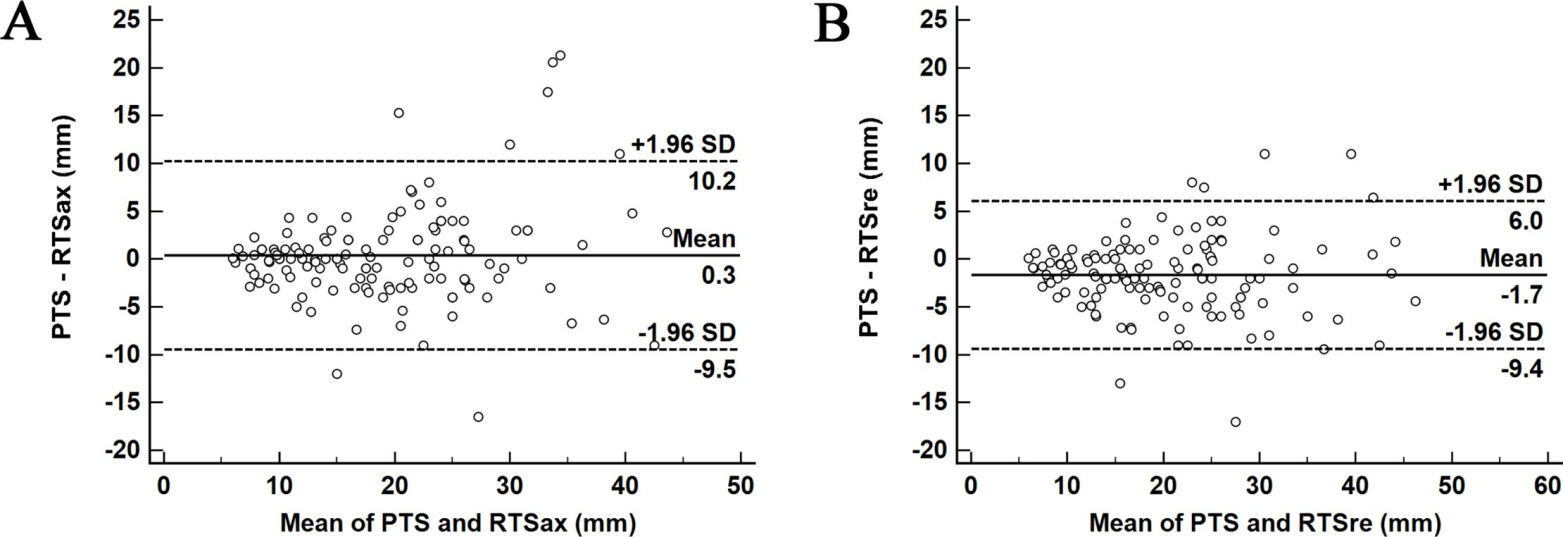
**Bland-Altman plots reflecting the differences between pathological tumor size (PTS) and radiological tumor size (RTS), which was measured in the axial image (A) and the multiplanar reformatted image (B).** The solid horizontal line indicates the mean difference between PTS and RTS; the horizontal dashed lines indicate the 95% limit of agreement.

The ICC between PTS and RTSax was 0.915 (95% confidence interval [CI], 0.881–0.940); and that ICC between PTS and RTSre was 0.954 (95% CI, 0.936–0.968). Bland-Altman plots are shown in Fig 3. The 95% limits of agreement were wider in RTSax (-9.5–10.2 mm) than in RTSre (-9.4–6.0 mm).

Independent *t*-tests revealed that pleural invasion (*p* = 0.047) or dimpling (*p* = 0.024) affected differences between PTS and RTSax (Table 3). The differences between PTS and RTSre were influenced by specimen type (*p* = 0.026), tumor location (*p* = 0.046), gross type in pathology specimens (*p* = 0.045), and pleural invasion (*p* = 0.047). Slice thickness in CT did not influence size discrepancies between PTS and RTS in simple linear regression analysis.

**Table 3:** Results of the independent *t*-test performed to investigate factors affecting differences between pathological and radiological tumor size.

Radiologic-pathological factors	n	Mean difference			
		PTS-RTSax (mm)		PTS-RTSre (mm)	
		Mean±SD or r	*p*	Mean±SD or r	*p*
Specimen type			0.081		0.026
†Partial resection	82	-0.3±4.3		-2.3±3.3	
‡Total resection	53	1.3±5.9		-0.8±4.7	
Location			0.362		0.046
Upper or middle lobe	87	-0.1±5.4		-2.2±4.1	
Lower lobe	48	0.8±4.3		-0.8±3.4	
Ischemic time (h)			0.300		0.225
≤24	119	0.1±4.9		-1.8±4.0	
>24	16	1.9±6.1		-0.6±3.6	
Gross type in pathology specimens			0.624		0.045
Solid or mucinous	80	0.7±4.8		-1.1±4.1	
Subsolid	55	-0.2±5.3		-2.5±3.6	
Pleural invasion			0.047		0.047
Absent	111	-0.1±4.5		-2.0±3.5	
Present	24	2.2±6.9		-0.2±5.4	
Pleural dimpling			0.024		0.086
Absent	81	-0.5±3.2		-2.2±3.1	
Present	54	1.5±6.7		-0.9±4.9	
Tumor necrosis			0.423		0.768
Absent	133	0.4±5.0		-1.7±3.9	
Present	2	-2.5±9.2		-2.5±9.2	
Histologic classification			0.258		0.863
AIS, MIA	15	-1.1±3.7		-1.9±3.8	
Invasive ADC	120	0.5±5.1		-1.7±4.0	
Lepidic predominant pattern			0.409		0.438
Absent	106	0.5±4.9		-1.5±4.0	
Present	29	-0.3±5.4		-2.2±3.9	
Slice thickness in CT (mm)		0.046	0.645	0.039	0.654

**†**Partial resection includes wedge resection and segmentectomy.

‡Total resection includes lobectomy and pneumonectomy.

PTS, pathological tumor size; RTSax, radiological tumor size measured on axial images; RTSre, radiological tumor size measured on multiplanar reformatted images; AIS, adenocarcinoma in situ; MIA, minimally invasive adenocarcinoma; ADC, adenocarcinoma; SD, standard deviation

Stepwise multiple linear regression (n = 135) was performed using radiological and histopathological factors with *p*<0.1 after the independent *t*-test: the input variables for discrepancies between PTS and RTSax were specimen type and pleural dimpling, and those for discrepancies between RTS and RTSre were specimen type, location, gross type in pathology specimens, and pleural invasion. Multiple linear regression analysis revealed that pleural dimpling (*p* = 0.024) was an independent factor affecting differences between PTS and RTSax (Table 4). Specimen type (*p* = 0.012) and tumor location (*p* = 0.020) were independent factors that resulted in differences in measurements between PTS and RTSre. After 90% random sampling of the study cohort using the SPSS software, we repeated multiple regression analysis, and found that beta and Pearson’s correlation coefficients were similar to those before random sampling (S1 Table).

**Table 4:** Multiple linear regression analysis performed to investigate independent factors affecting differences between pathological and radiological tumor size.

Parameters	PTS-RTSax		PTS-RTSre	
	B (SE)	*P*	B (SE)	*P*
Pleural invasion: Present vs. absent (reference)				
Pleural dimpling: Present vs. absent (reference)	1.98 (0.868)	0.024		
Specimen type: †Total resection vs. partial resection (reference)			1.731 (0.678)	0.012
Location: Lower lobe vs. Upper or middle lobe (reference)			1.624 (0.692)	0.020
Gross type in pathology specimens: Subsolid vs. solid or mucinous (reference)				

†Total resection includes lobectomy and pneumonectomy; partial resection includes wedge resection and segmentectomy.

PTS, pathological tumor size; RTSax, radiological tumor size measured on axial images; RTSre, radiological tumor size measured on multiplanar reformatted images; SE, standard error

**Fig 4:**
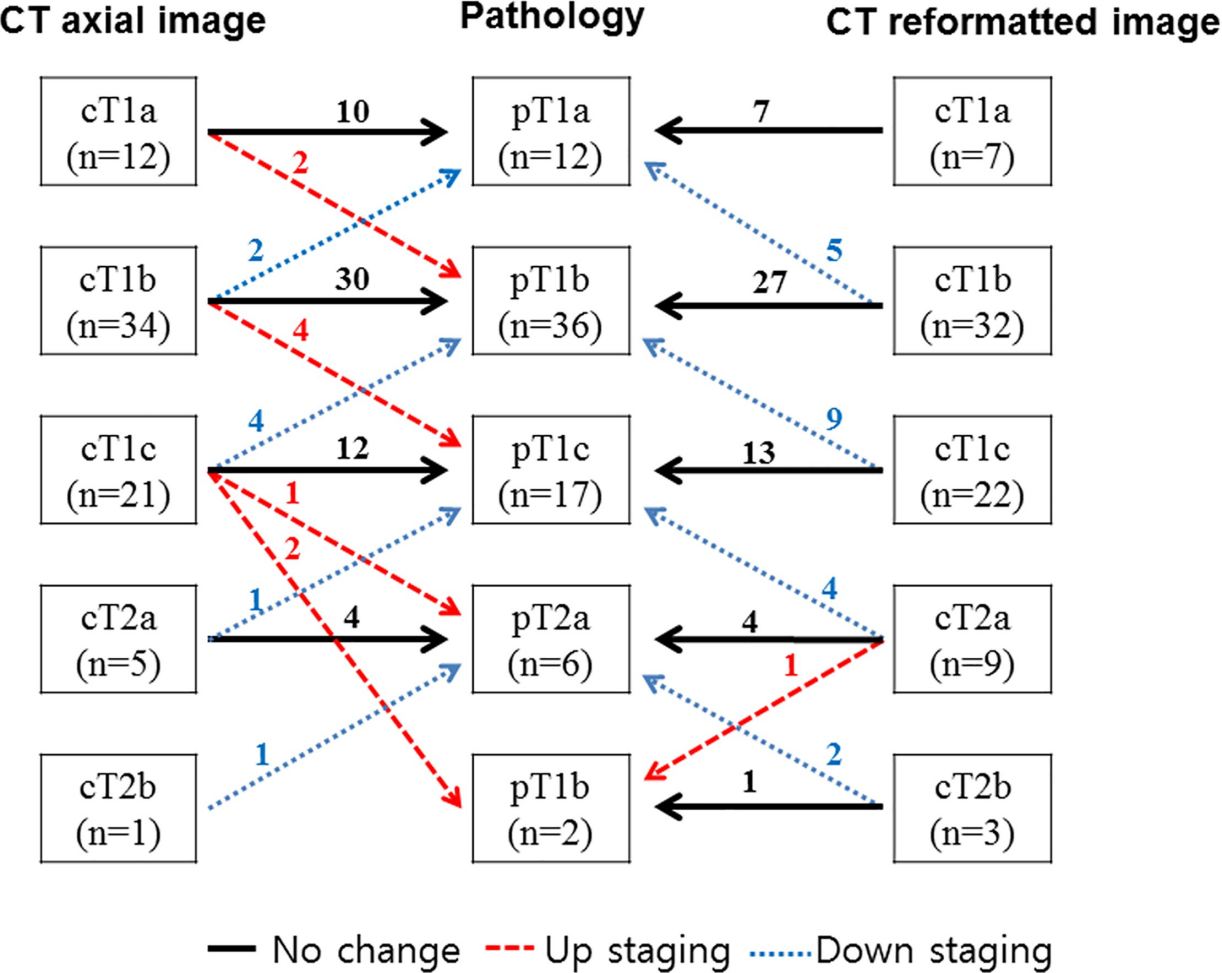
Changes in T stage after operation. T staging before operation was performed using radiological tumor size, which was measured on axial images and the multiplanar reformatted CT images and then compared with pathological tumor size.

Among the 73 invasive adenocarcinomas that did not exhibit pleural invasion, nodal metastasis, and lepidic predominant pattern, there was a discrepancy between radiological and pathological T staging in 17 (23.3%) and 21 (28.8%) tumors when radiological staging was performed using RTSax and RTSre, respectively (Fig 4, Table 5). Of these, 8 (11.0%) tumors were down-staged and 9 (12.3%) were up-staged postoperatively when radiological staging was performed using RTSax. However, 20 (27.4%) tumors were down-staged postoperatively and 1 (1.4%) was up-staged when radiological staging was performed using RTSre. No cancer-related death was observed in this subpopulation. Three cases of cancer recurrence were observed during follow-up (median, 45 months; range, 0–63 months), but there was no statistically significant difference in patient outcome among T stages performed using PTS, RTSax, and RTSre.

**Table 5:** Discrepancy between radiological and pathological tumor stage in 73 invasive adenocarcinomas.

	Pathological T stage^a^ (n)	Radiological T stage^a^		Down staged after operation		Upstaged after operation	
		RTSax (n)	RTSre (n)	RTSax (n)	RTSre (n)	RTSax (n)	RTSre (n)
T1a	12	12	7	2 cT1b to pT1a	5 cT1b to pT1a		
T1b	36	34	32	4 cT1c to pT1b	9 cT1c to pT1b	2 cT1a to pT1b	
T1c	17	21	22	1 cT2a to pT1c	4 cT2a to pT1c	4 cT1b to pT1c	
T2a	6	5	9	1 cT2b to T2a	2 cT2b to pT2a	1 cT1c to pT2a	
T2b	2	1	3			2 cT1c to pT2b	1 cT2a to pT2b
Total	73	73	73	8 (11.0%)	20 (27.4%)	9 (12.3%)	1 (1.4%)

^a^Stages adopted from the American Joint Committee on Cancer TNM staging system, 8th edition

RTSax, radiological tumor size measured on axial images; RTSre, radiological tumor size measured on multiplanar reformatted images

## Discussion

Tumor size became a very important prognostic descriptor for NSCLC in the American Joint Committee on Cancer Staging Manual, 8^th^ Edition[14]. For T staging purposes in lung cancer, the manual defines RTS as the single largest tumor dimension measured on axial, coronal, or sagittal CT sections at the lung window setting[14, 15]. Measurement of PTS is recommended to be performed in fresh specimens after cross-sectioning[9, 16]. As the longest tumor axis does not always align with axial, coronal, or sagittal planes, we also evaluated the RTS in obliquely reconstructed CT images in this study. In addition, as measuring tumor size in a cut section of fresh lung tissue is not a routine practice in pathology, we specifically designed the study cohort to measure PTS in the fresh state using the same cutting methodology.

A previous study had compared the utility of multiplanar reformatted CT and pathology; Pawaroo *et al*. showed that multiplanar CT measurement at lung window setting overestimates PTS (it is not clear whether the specimen’s state was fresh or formalin fixed) with a mean difference of 7.8 mm in NSCLC[17], which is much larger than that observed in our study.

Recent studies have revealed discrepancies between preoperative RTS measured using axial CT section and postoperative PTS in small lung adenocarcinoma. Lampen-Sachar *et al*. reported that axial CT measurement was significantly larger than pathological measurement in the fresh state, with a mean difference of 5.49 mm[5]. Conversely, Heidinger *et al*. demonstrated that axial CT diameter was significantly smaller than pathological measurement in the fixed state, with a mean difference of 0.8 mm in case of solid nodule[6]. In case of pure ground-glass nodule, Heidinger *et al*. showed that axial CT diameter was larger than pathological measurement in the fixed state[11]. In contrast, Isaka *et al*. reported that RTS measured on axial CT section was similar to PTS measured in inflated fresh tissue section after saline immersion[8]. Bhure *et al*. also showed that RTS measured on axial CT image was similar to PTS measured on axial histological section[10]. These conflicting findings may result from differences in study populations (proportions of solid and subsolid masses), method of tumor delineation in CT (semi-automated vs. manual), slice thickness in CT, and tissue preparation. Specimen preparation, such as tissue section immersion and cutting methodology, may differ according to institution and gross prosecutors, which can lead to discrepancies in PTS. Tissue section immersion with saline has a slight expansion effect on lung tissue and subsolid masses, and could result in increases in PTS. Unlike CT measurement, specimen cutting is a one-time procedure, and PTS cannot be measured repeatedly. In tertiary referral hospitals, gross cutting is usually performed by different pathology residents, which may introduce bias in retrospective studies. To reduce interobserver variability when measuring PTS in this study, specimen cutting and tumor size measurement was performed in the fresh state by one staff pathologist specializing in pulmonary pathology.

Given that the lung is inflated during CT examinations and RTS is larger when the greatest tumor diameter is measured on multiplanar reformatted CT images than on axial CT images alone[18], it is reasonable to conclude that multiplanar reformatted CT measurement overestimate pathological measurement. Herein, we demonstrated that RTSre was significantly larger than PTS; RTSax was not statistically significantly different from PTS in this study, which is consistent with the findings of a previous study by Isaka *et al*[8].

Both RTSax and RTSre were significantly larger than PTS in tumors ≤2 cm, and the mean difference was greater in RTSre than in RTSax. In small adenocarcinoma, chest CT tended to overestimate PTS, regardless of the plane; however, multiplanar reformatted CT overestimated PTS more than axial CT (mean difference, 2.3 mm vs. 1.1 mm). This result can be explained by the increased partial volume effect for smaller nodules[19] and reformatted images[18]. Among the tumors larger than 4cm in PTS (n = 6), 4 had vertically oriented tumor axis, and this may cause greater underestimation of PTS by RTSax than by RTSre (mean difference, 13mm vs 2.3mm).

In this study, RTSre was found to be more strongly correlated with PTS than RTSax. Most lung tumors have spiculated and geographical margins rather than a spherical shape, and the long axis of the tumor does not always align with the transverse CT plane; consequently, reconstructed CT planes are more relevant to the largest tumor section in specimens. In fact, it is impossible to equate radiological tumor images with pathological tumor sections, especially in wedge resection specimens, because the three-dimensional shape of the lung tissue changes even after the removal of surgical staples. Tumor sections in pathology specimens occasionally cannot reflect the actual largest tumor cross section because tumors cut perpendicular to the visceral pleura to evaluate pleural invasion of tumor in case of tumors exhibiting pleural puckering.

Independent *t*-tests and simple linear regression analysis revealed that pleural invasion and dimpling are factors that influence differences in RTSax and PTS. Factors, such as specimen type, location, gross type in pathology specimens, and pleural invasion influence differences in RTSre and PTS. The lepidic component of adenocarcinomas is believed to be responsible for tumor contractibility during formalin fixation, slide preparation, and paraffin embedding. However, it did not affect tumor contractibility between radiology results and fresh pathology specimens in this study. Multiple linear regression analysis revealed that pleural dimpling was an independent factor affecting differences in RTSax and PTS, while specimen type and location were independent factors influencing differences in RTSre and PTS. Although pleural dimpling was found to preserve tumor size during formalin fixation, it increased the size difference between axial CT measurements and PTS in this study. We were not able to discern why partial resection, and upper or middle lobar location of the tumor, increased the size discrepancy between PTS and RTSre.

Tumor stage changes between RTS and PTS were evaluated, and survival analysis were performed only in invasive adenocarcinomas that did not exhibit pleural invasion, nodal metastasis, and a lepidic predominant pattern. Since RTSre tends to overestimate PTS, and RTSax is not statistically different from PTS, postoperative down-staging was more frequent (27% vs. 10.8%) when radiological tumor staging was performed using multiplanar reformatted images (i.e., RTSre) than axial images (i.e., RTSax). Tumor stage discrepancy between RTS and PTS did not affect prognosis. In the future, it is necessary to study whether RTS or PTS are more related to patient outcome in NSCLC with a large sample cohort.

This study has some limitations. Size comparisons between radiological and pathological methods were performed for the entire tumor size, including the lepidic (noninvasive) component. It is often difficult to discriminate invasive adenocarcinoma from the lepidic component on gross examination. Papillary predominant invasive and acinar predominant invasive adenocarcinoma with less fibrous stroma can present with a subsolid mass. There is a “gray zone” mass between compacted solid and subsolid tissue on macroscopic examination. In addition, the sample size in this present study might have been insufficient for in-depth analysis, but we think this study make significant contributions because the study cohort was obtained from prospectively collected lung cancer data, unlike other retrospective studies[5, 6, 10–12, 17]. The statistical power is inadequate for tumor size discrepancy between pathology and radiology in this study, which paradoxically indicates that PTS and RTS might not be significantly different on average.

In conclusion, radiological tumor measurement performed using multiplanar reformatted images overestimates fresh PTS in lung adenocarcinoma, with 27.4% of the tumors exhibiting postoperative down-staging. In adenocarcinomas ≤20 mm, axial CT measurement also overestimates PTS. Multiplanar reformatted CT measurement correlate better with PTS than axial CT measurement. Partial resection and upper or middle lobar location of the tumor, may increase the size discrepancy between PTS and RTSre.

## Supporting information

S1 Dataset (XLS)

S1 Table. Comparison of multiple regression analysis between whole and 90% random sampling data (DOCX)
