# Supplementary material for: Correlation between maximal tumor diameter of fresh pathology specimens and computed tomography images in lung adenocarcinoma
Source: PLoS One. 2019 Jan 25;14(1):e0211141. doi: 10.1371/journal.pone.0211141 (PMC6347433; doi:10.1371/journal.pone.0211141)
Supplement: S1 Table. Comparison of multiple regression analysis between whole and 90% random sampling data — (DOCX) [file pone.0211141.s002.docx]

**S1 Table. Comparison of multiple regression analysis between whole and 90% random sampling data**

|  | Data set | PTS-RTSax | | | | PTS-RTSax | | | |
| --- | --- | --- | --- | --- | --- | --- | --- | --- | --- |
|  |  | B (SE) | *P* | Beta | Pearson’s correlation  coefficient | B (SE) | *P* | Beta | Pearson’s correlation  coefficient |
| Pleural invasion |  |  |  |  |  |  |  |  |  |
| Pleural dimpling | Whole (n=135) | 1.977 (0.868) | 0.024 | 0.194 | 0.037 |  |  |  |  |
|  | 90% sampling (n=121) | 1.867 (0.902) | 0.041 | 0.183 | 0.034 |  |  |  |  |
| Specimen type | Whole (n=135) |  |  |  |  | 1.731 (0.678) | 0.012 | 0.215 | 0.075 |
|  | 90% sampling (n=121) |  |  |  |  | 1.824 (0.690) | 0.009 | 0.234 | 0.066 |
| Location | Whole (n=135) |  |  |  |  | 1.624 (0.692) | 0.020 | 0.198 | 0.075 |
|  | 90% sampling (n=121) |  |  |  |  | 1.447 (0.690) | 0.038 | 0.186 | 0.066 |
| Gross type in specimen |  |  |  |  |  |  |  |  |  |

PTS, pathological tumor size; RTSax, radiological tumor size measured on axial images; RTSre, radiological tumor size measured on multiplanar reformatted images; SE, standard error
